# Closed-loop neuromodulation will increase the utility of mouse models in Bioelectronic Medicine

**DOI:** 10.1186/s42234-021-00071-x

**Published:** 2021-06-30

**Authors:** Timir Datta-Chaudhuri

**Affiliations:** 1 Institute of Bioelectronic Medicine, The Feinstein Institutes for Medical Research, Northwell Health, 350 Community Drive, Manhasset, NY 11030 USA; 2 Donald and Barbara Zucker School of Medicine at Hofstra/Northwell, 500 Hofstra University, Hempstead, NY 11549 USA

**Keywords:** Bioelectronic medicine, Implantable devices, Neuromodulation, Mouse model, Preclinical research, Engineering challenges, Vagus nerve stimulation

## Abstract

Mouse models have been of tremendous benefit to medical science for the better part of a century, yet bioelectronic medicine research using mice has been limited to mostly acute studies because of a lack of tools for chronic stimulation and sensing. A wireless neuromodulation platform small enough for implantation in mice will significantly increase the utility of mouse models in bioelectronic medicine. This perspective examines the necessary functionality of such a system and the technical challenges needed to be overcome for its development. Recent progress is examined and the outlook for the future of implantable devices for mice is discussed.

## Introduction

Bioelectronic medicine (BEM), using neuromodulation to control physiological homeostasis and diagnose and treat diseases, is a nascent but rapidly growing field. Since the discovery by Tracey and others on the implications of vagus nerve signaling on the immune system (Pavlov & Tracey, 2012), many researchers have shown interest in studying the potential therapeutic effects of peripheral nerve stimulation. Unlike other more established fields of research, the delivery and evaluation of BEM therapies does not yet have a standardized set of approaches or tools. This has resulted in different research groups developing their own approaches and devices for their indications of interest. Further, the interdisciplinary nature of BEM requires expertise in not only the particular indication of study but also in neural engineering, electrical engineering, neural interfaces, neurophysiology, mechanical engineering, materials science, and other fields, raising the bar for entry into the field. For these reasons, progress is outpaced by demand, and potential opportunities for BEM applications remain unexplored or stagnant. A widely available generalizable, indication-agnostic, neuromodulation platform with modular capability, that can be adopted by researchers without the need for significant custom development will address this gap.

It can be argued that the laboratory mouse has had the greatest impact on the advancement of modern medicine. The mouse model has been the starting point of choice for many researchers in medical science since the 1920s (Fox et al., 2006). Numerous models of disease exist for the mouse, and techniques for establishing new models are mature enough to enable rapid developments for new targets (Silverman et al., 2018). Though most BEM researchers do use mouse models for their studies, this research has mostly been limited to acute experiments consisting of a few minutes to a few hours of intervention (Tsaava et al., 2020; Caravaca et al., 2019; Meneses et al., 2016). The lack of broad access to tools to move beyond such acute experiments has meant that it has not been possible to study the effects of BEM interventions in mice over biologically relevant time periods of weeks to months. Developing a standardized set platform for studying BEM in mice, may very well be the single most important advancement required for the continuing progress in the field. The mouse, however, presents challenges for implementation of these approaches due to its size. Anatomical targets (brain, peripheral nerves, and organs) in mice are often smaller than available technology for interfacing with similar targets in other models, and importantly, though neuromodulation technology has matured significantly in capability over the last few decades, most developments have not been miniaturized to a scale that is readily applicable to mice.

This perspective will examine some of the important areas of progress that are required to advance the utility of mouse models in BEM. Specifically, the ability to perform interventions chronically instead of acutely, the added value of wireless fully implantable neuromodulation systems, the importance of closed-loop approaches, the technical challenges in these areas, and lastly a look at the new opportunities arising from integrating bio-sensing in addition to bio-potential sensing. Though there are numerous targets and approaches (Montgomery et al., 2015; Shin et al., 2017; Gutruf et al., 2019; Yang et al., 2021; Huerta et al., 2021; Cotero et al., 2019) for neuromodulation in BEM, electrical stimulation of the vagus nerve is of particular interest because of its broad connectivity and relative ease of access at the cervical location. Vagus nerve stimulation (VNS) has been approved by the FDA for treatment of epilepsy and depression (Ben-Menachem, 2002) and there are efforts underway to discover new clinical and basic scientific research applications of VNS (George et al., 2000; Gold et al., 2016). Advances, challenges, and opportunities will be discussed within the context of VNS in mice, with the understanding that developing electronic devices capable of VNS requires overcoming similar technical challenges as other neuromodulation methods (optical, mechanical, etc.) while requiring only incremental changes to enable these other approaches.

## Chronic interventions

Neural interfaces for the brain of the mouse exist that are capable of recording and stimulation over chronic durations of time (Moxon et al., 2004; Mols et al., 2017; Juavinett et al., 2019). Tools for recording from the central nervous system (CNS) are mature and capable of recording from volumes of neural tissue down to single neurons in rodents (Mols et al., 2017; Chung et al., 2019; Xu & Wilson, 2012). Similarly, there exist a variety of approaches for electrical and optical stimulation (Lim et al., 2013) of the CNS. Devices for interfacing with the mouse brain have the advantage that they can be secured to the skull, and relative movement between the brain and skull can be negligible, allowing robust performance over long periods of time.

Neural interfaces for the peripheral nervous system (PNS) are not as advanced. The vagus nerve is an attractive target for BEM because it serves as a trunk location containing fibers that subsequently access different anatomical loci. Though the vagus nerve is one of the larger nerves, it is usually not larger than 100 μm in diameter at the cervical level in mice, and it is also surrounded by soft tissues which make it impossible to anchor interfaces to the same degree as is possible in the brain. Correspondingly, vagus nerve electrodes are highly susceptible to motion artifacts that show up in recordings (Zanos et al., 2018; Steinberg et al., 2016). They are also subject to interference by other bio-potentials such as ECG and EMG. For these reasons, chronic recordings from the mouse vagus have not yet been shown to be viable over long durations of time. Similarly, attempts to chronically stimulate the mouse vagus nerve suffer from the foreign body response (FBR), resulting in local inflammation and ingress of encapsulating tissue between the electrode and the nerve after implantation (Mughrabi et al., 2021). Stimulation thresholds rise, and electrode impedances increase, placing greater burden on the stimulation system.

Ideally, neural interfaces to the mouse PNS should last indefinitely. Progress towards this goal will require concurrent advances in electrode design and surgical approaches to better manage the FBR after implantation. Recent developments indicate promising progress in the development of chronic stimulation electrodes for the vagus nerve in mice (Mughrabi et al., 2021), which have been shown to be able to elicit a bradycardia response to stimulation over multiple weeks. Chronic recordings from the mouse PNS remain an outstanding challenge. Unlike stimulation, where a physiological response can be used to determine stimulation efficacy, ground truth (to differentiate true neural signals from noise) does not exist for recorded spontaneously occurring neural signals. One way to circumvent this issue is to record evoked potentials that arise in response to stimulation, but this requires co-location of both types of electrodes which in itself can be a challenge because of the limitations on size. Recording evoked potentials introduces another issue of stimulation induced artifacts, requiring mitigation strategies to reject them or to compensate for their effects while also necessitating careful design of the electronics to protect amplifier inputs from stimulation voltages (Chandrakumar & Markovic, 2017).

Moving beyond acute studies is critical to the translation of BEM therapies. Though chronic neuromodulation can be more readily implemented in large animal models, the availability of existing mouse models of disease and the ease of developing new models using mature genetic and pharmacological approaches makes the mouse compelling for research in BEM. Additionally, many BEM researchers are immunologists or biologists that already employ mouse models, and the mouse presents a low barrier for entry for new researchers compared to large animals or clinical studies. Furthermore, several important discoveries on vagus nerve mediated immune responses implicated in numerous diseases have arisen from acute stimulation or vagotomy experiments (Borovikova et al., 2000a; Borovikova et al., 2000b; van Westerloo et al., 2006; Huston et al., 2007) over the last two decades, yet clinical translation of these findings is lacking. Chronically implanted devices for mice will enable not only the delivery of long-term targeted stimulation and reversible vagotomy through the use of techniques such as fiber selective waveforms and high-frequency blocking (Pelot & Grill, 2018), but will allow researchers to study the effects of interventions over the course of disease progression and recovery.

## Wireless fully implantable systems

Current approaches for chronic stimulation and recording in mice, whether for the CNS or PNS, mostly rely on wired connections. A wireless fully implantable approach integrates stimulation and sensing capability, along with other features, into a single device that resides within the body. The core advantages of such devices are that they can be used in awake untethered freely behaving mice, removing the effects of restraint, anesthesia, and animal handling from biasing the experimental findings.

**Fig. 1:**
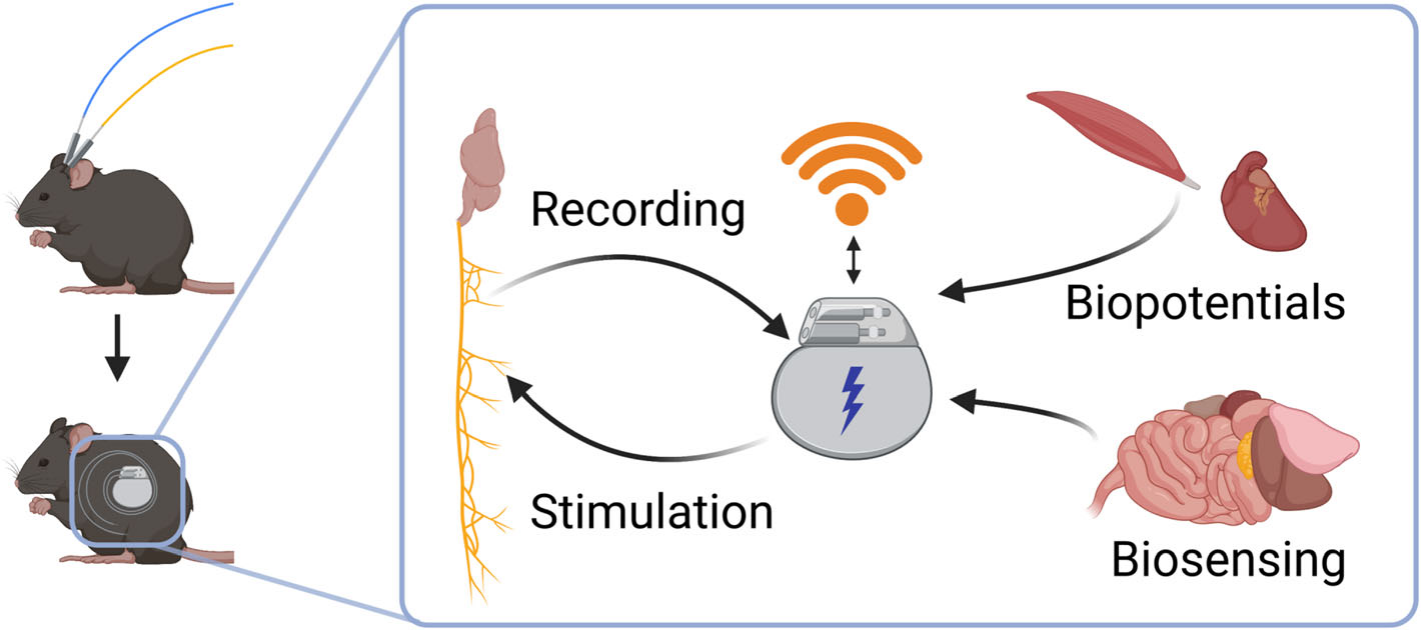
Transition from tethered approach to a fully implantable approach. The implantable device provides stimulation and sensing capability. Data telemetry and control signals are communicated using a wireless interface

Implementation of a wireless fully implantable system presents many technical challenges which are discussed in detail later on. The core functionality of a generalizable fully implantable system is shown in Fig. 1, where it can be seen that the wired interface is replaced by an implanted system capable of stimulation/sensing, and energy storage and wireless communication. Prior efforts to develop neuromodulation systems for small animals have had varying degrees of success. One approach has been to use partially implantable systems consisting of components both inside and outside the body, with externalized head stages that are connected to implanted neural interfaces (Juavinett et al., 2019; Alpaugh et al., 2019). Such percutaneous devices introduce additional potential issues arising from longer healing time post-surgery, increased likelihood of subsequent infection, and the possibility that the protruding structure can be damaged by the environment or the mouse itself. Other approaches small enough to be implanted in mice are limited in their capabilities, sometimes only providing stimulation (Talkachova et al., 2013; Millard & Shepherd, 2007; Madan et al., 2020) or requiring external power delivery during operation (Piech et al., 2020). Larger devices appropriate for implantation in rats have been developed (Lee et al., 2019; Liu et al., 2015), but cannot be used in mice. Some commercial devices for implantation in mice exist, but are limited to sensing pressure, respiratory rate, movement, or in some cases low sampling rate biopotentials such as ECG or EMG (DSI Harvard Bioscience). Interestingly, a commercial implantable stimulator for mice (Deshmukh et al., 2020) was previously available (TBSI Harvard Bioscience), but appears to no longer be in production.

Many of the technical challenges for developing wireless fully implantable systems for mice can be reduced or eliminated by using battery-free devices, which have recently been used for optogenetic stimulation (Mickle et al., 2019), electrical stimulation (Piech et al., 2020), and sensing of various biomarkers (Won et al., 2021). Beyond removing the battery, these devices achieve the required level of integration and miniaturization by limiting the feature set to that which is required for a specific application. Inherently, these devices are application specific. Development of a generalizable system, with flexible stimulation and sensing capabilities, is critical to enable adoption by a broad group of researchers.

## The need for closing the loop

Most clinical neuromodulation therapies are performed in a primarily open-loop manner. Stimulation is applied based on parameters known to be safe and effective based on prior findings. In some cases, like deep brain stimulation or spinal cord stimulation, adjustments are made for optimizing subject-specific response. This tuning is usually performed by a clinician at the time of implantation or at specific time points thereafter. This clinician-in-the-loop methodology is more effective than a purely open-loop approach, but it does not allow for optimizing of stimulation over the entire course of the intervention. The starting points for stimulation parameters are based on preclinical findings and prior clinical safety and efficacy studies. Approaches for therapy specificity in these initial studies include electrode design and placement, and the use of stimulation waveforms that are designed to be fiber selective. Though the techniques for fiber selectivity are well established theoretically, in practice they depend on anatomical variability between subjects and species to species variations, which further detract from the ability to perform selective and target specific stimulation. The result of this is that stimulation parameters which worked well in a particular subject or species do not translate optimally to others. Additionally, changes in electrode position and the FBR can play a role in decreasing efficacy within an individual over time. The implications of this are broad and are likely the underlying cause of failed clinical trials in VNS and other BEM applications (De Ferrari et al., 2017).

Closed-loop approaches offer the promise of avoiding such issues by enabling real-time assessment of stimulation therapy efficacy and adjustment to optimize the response to affect a particular outcome. The need for closed-loop approaches in clinical application is well recognized (Keaney et al., 2017), but identification of appropriate biomarkers remains a challenge (Hell et al., 2019; Morishita & Inoue, 2017). Implementing closed-loop systems for preclinical studies, especially for the many existing mouse models of diseases, presents an opportunity to not only develop the technology and techniques, but also to identify appropriate biomarkers for clinical translation. Closed-loop neuromodulation requires the incorporation of sensing in addition to stimulation, the readout can be electrical (neural or physiological), mechanical (pressure, motion, etc.) or chemical (inflammatory, metabolic, etc.). Also required is some degree of evaluation and assessment that is performed either on-board the device itself or possibly using a computer. Implementing closed-loop adds significantly to the level of integration required to produce such systems, but the benefits may very well outweigh the cost.

## Technical challenges

The mouse model presents several technical challenges to implementation of wireless implantable neuromodulation systems, these challenges largely stem from the diminutive size of the mouse. Without specific guidance, most engineers rely on IACUC regulations (stemming from guidance provided by AAALAC (Workman et al., 2010), the NIH (National Institutes of Health OACU, 2019), and the USDA (Allen & Kreger, 2000) for tumor size and mass in rodents. These sources generally indicate that implants should not significantly exceed 10% of body mass and that the largest dimensions remain below 2 cm. As a result of these constraints, it is impossible to leverage the many of the advances in neuromodulation technologies that have emerged from clinical device development.

The components of a fully implantable neuromodulation system for mice include electronics for interacting with biology such as stimulus generators and recording amplifiers, interfaces such as electrodes for transducing between the electrical and biological domains, a computational component, a communication module for telemetry and control, a power supply, and encapsulation or packaging to protect the electronics from the biology and vice versa. The design and implementation of each of these components is intertwined with the requirements and execution of the other components, necessitating a systems-level approach to the design of implantable devices.

### Front-end design

The electronics and interfaces that interact with biology are collectively known as the “front-end” of a neuromodulation system. Front-end components include stimulators, biopotential sensors, and electrodes. Advances in semiconductor fabrication driven by the personal computer industry have enabled integration of dense and complex electronic functionality into very small areas. Neural stimulation and recording electronics have shrunk to nearly the size of individual neurons (Muller et al., 2015). In fact, the same advances from the semiconductor industry have resulted in microfabricated electrodes that are on par with the scale of individual neurons (Vahidi et al., 2020). Using these fabrication techniques, electrodes and electronics can be co-located (Datta-Chaudhuri et al., 2016; Datta-Chaudhuri et al., 2014a; Datta-Chaudhuri et al., 2014b), but these approaches are generally not appropriate for chronic implantation as they lack robust barrier layers. Microfabricated electrodes have been developed for CNS applications in small animals using both silicon (Massey et al., 2019) and polymer substrates (Chung et al., 2019), but wires or traces must be used to connect isolated electronics from the device body to the electrode contacting the nervous system tissue. When developing interfaces for the PNS the form factor of the electrode body including cuffs, arrays, and piercing structures require non-planar structures, and the microfabrication techniques used to produce exquisitely intricate two-dimensional structures do not always translate well to three dimensions. As a result of this, many PNS electrodes have a significant handmade component, a challenging task for the small anatomical targets in mice.

Sensing of bio-potentials has seen tremendous advancement as implants have developed from a technology perspective. Numerous amplifiers and electrodes have been developed to sense electrical signals such as neural action potentials, signals from muscles (EMG), and from the heart (ECG) (Muller et al., 2015; Zhou et al., 2018; Reich et al., 2021). While EMG and ECG signals are large and easy to sense and therefore identify, neural signals outside of the brain can be difficult to sense over long durations with chronically implanted electrodes. This issue is even more pronounced for the mouse since in many cases peripheral nerves of interest are measured in double digit micrometers (Le Pichon & Chesler, 2014) and often hit the limits of electrode fabrication capability (Caravaca et al., 2017). Reliable sensing of peripheral nerve signals in mice and other small animals remains an ongoing area of development in pre-clinical research.

Increasing component density for electrodes and electronics adds additional challenges beyond the physical constraints. As channel count increases so does the amount of data that needs to be processed and transmitted, requiring correspondingly more power, computational capability, and higher rate data telemetry. Another side-effect of increasing channel count in a constrained space is that the area of individual electrodes gets smaller, this combined with the FBR results in high electrode impedances which can continue to increase over time (Cassar et al., 2019). Higher impedances require higher voltages to deliver a given amount of current, placing greater demand on stimulation electronics. These high-voltage circuits typically take up more room and operate at lower efficiency, which in turn limits space available for other features and consumes more energy. So, while it is theoretically possible to integrate numerous electronic and electrode channels, practicality of application limits the real-world implementation.

Fortunately, significant progress has been achieved in the development of electrode materials for efficient stimulation, decreasing the burden on stimulation electronics. Electrode materials are characterized by their ability to safely deliver charge while avoiding irreversible electrochemical reactions at the electrode surface that can degrade the electrode and produce toxic byproducts (Cogan, 2008; Cogan et al., 2016). The water window, defined as the reduction and oxidation potentials of water for a given electrode material is one way to define the limits of safe stimulation. A related metric is charge injection capacity (CIC), specifying the amount of charge an electrode can deliver for a given area while remaining within the water window. However CIC limits can vary depending on current pulse durations and amplitudes, with various groups reporting different CIC values for similar materials when testing conditions differed (Cogan, 2008). Electrode impedance is loosely inversely proportional to area and CIC (Cogan, 2008), with larger lower impedance electrodes requiring lower voltages to deliver a given amount of charge over a period of time compared to smaller electrodes. Platinum, which is often alloyed with Iridium, is commonly used for clinical applications and is recognized to be durable and safe over long periods of time. Platinum however, has a relatively low CIC (Leung et al., 2015), other emerging materials such as various forms of Iridium Oxide and poly (3,4-ethylenedioxythiophene) (PEDOT) have CIC values of roughly 20x to 100x (respectively) as that of Platinum (Cogan, 2008), and are good candidates for the microelectrodes required for accessing targets in mice. Validation of the durability of these materials is ongoing (Straka et al., 2018; Dijk et al., 2019).

### Supporting electronics

Components for computation and communication are the supporting electronics necessary achieve system-level functionality. They are used to perform on-board signal processing, execute algorithms for closing the loop, and transmit data and commands between the implant and controller such a computer. Many of these functions can implemented in custom integrated circuits (ICs) which incorporate the front-end electronics. Although these areas lie within electrical engineering, the sub-disciplines are very different, and require a diverse design team to build such integrated systems. Industry offerings specifically designed for implantable neuromodulation are practically non-existent, meaning that advances in this area generally arise out of academic laboratories resulting in limited availability and support. Often, different components from different sources are brought together to build a system, a front-end IC from one source, a processor from another source, and a wireless interface such as a Bluetooth module from yet another source. The implications of this are that systems are larger than they must be, consume more power than fundamentally necessary, and require greater energy storage or transfer. Systems-on-chip developed by industry, that incorporate the supporting electronics functionality into a single component, will provide the necessary solution to this need. Thankfully, development of such a device is well within the capability of the major electronics manufacturers.

### Power and packaging

The power supply and the biocompatible encapsulation are linked because stored electrical energy necessitates robust isolation from biological fluids. The presence of electrical potentials drives electrochemical reactions that can result in component degradation and failure, potentially producing harmful byproducts within the body. Systems that do not store energy also require encapsulation since internal components of the device can be toxic to biology, and the ingress of conductive biological fluids can lead to similar failure mechanisms while the device is powered.

No matter how small electronics can be made, one of the major components of an implant which cannot be scaled easily to mouse proportions is the battery. In fact, up to 80% of the volume of clinical implants is taken up by the battery (Amar et al., 2015). As energy storage devices, batteries have certain structural and functional components that do not scale well, resulting in significantly lower stored energy per volume as battery sizes are reduced. Battery-less devices circumvent the issue of added battery volume but can add additional requirements. Ultrasound and electromagnetic energy transfer are two popular approaches to either eliminate batteries or to allow the use of smaller batteries. Ultrasound-based power transfer (Piech et al., 2020) requires the use of a transducer in contact with the skin, necessitating animal handling and conditioning. Electromagnetic wireless power transfer can employ different techniques including inductively coupled systems (Lee et al., 2019) and novel magnetoelectric materials (Singer et al., 2020) but these systems must consider the interaction of the electromagnetic waves with tissue. There are limits for human exposure to electromagnets waves specified in terms of specific absorption rate (SAR), but it’s not clear how and if these apply to freely moving small animals (Chen et al., 2017), or if the effects are exacerbated when the source of the wireless signal is within the body (Helwig et al., 2012) when wireless telemetry is used. Regardless of how the device is powered, designers must consider the heat generated by the operation of the electronics. Depending on the level of blood flow through a volume of tissue the limits for safety range up to +/− 2 degrees Celsius (Reichert, 2007). Power dissipation limits for implants are provided in terms of power per package surface area (Datta-Chaudhuri et al., 2016). Increasing the efficiency of the electronics and reducing the requirements for data telemetry can reduce power consumption, correspondingly reducing heat generation, battery size, and energy transfer requirements.

Packaging devices for implantation is a major challenge for small animal models. Clinical implants utilize metals and ceramics as barrier layers. Both metals and ceramics can be biocompatible and have extremely low water vapor transmission rates (WVTR) (Shen & Maharbiz, 2021). Clinical implants can last for decades, but the same characteristic that makes these materials ideal for clinical devices (namely high density) detracts from its use for small animals. Metals and ceramics fabricated using current approaches weigh too much to be used for mouse implants. Additionally, the costs associated with the design, manufacture, and validation of new metal/ceramic enclosures is very high and may exceed resources available at most research laboratories.

Thin-film technologies and polymers offer a viable alternative for traditional packaging approaches. Thin-films such as silicon dioxide (Fang et al., 2016), hafnium oxide and alumina (Selbmann et al., 2017; Ahn et al., 2019), can be deposited in nanometer thick layers and offer excellent barrier properties. However, the deposition processes for these materials are not always compatible with other system components (Fang et al., 2016), and mechanical robustness is limited due to their brittle nature (Gaskins et al., 2017). Nevertheless, thin-film encapsulation techniques show tremendous promise for current and future applications. Access to the tools and specialized equipment required for thin-film fabrication is already available at many university facilities in their micro and nano fabrication facilities that are used for teaching and other research activities.

Polymers are an alternative approach for developing barrier layers that are generally low cost and do not require overly specialized manufacturing processes. The downside to polymers is that they have orders of magnitude higher WVTR (Hogg et al., 2014). One approach to combat this is to combine different layers to benefit from the strengths of each. Popular polymers include Parylene-C (Loeb et al., 1977), epoxies (Wright et al., 2019), and liquid crystal polymers (LCPs) (Gwon et al., 2016). From a high-level system design approach, it is important to recognize that implants for small animals do not need to last for decades, so a semi-hermetic approach using polymers may meet experimental requirements (Boeser et al., 2016). Additive manufacturing processes for polymers, such as 3D printing, are recently becoming widely available and have been used to successfully package implantable devices (Kölbl et al., 2014; Yu et al., 2020). If shown to be viable over appropriate time durations, these approaches may help to significantly ease implantable package design.

## Sensing beyond bio-potentials

One of the attractive promises of BEM is that information about the immune and metabolic state are contained in the electrical signals of the nervous system. But decoding such information requires understanding the neural code at a far greater level than what is currently understood. Neural decoding is an ongoing area of research employing techniques such as machine learning and artificial intelligence to try to elucidate meaning from electrical recordings (Masi et al., 2019). Until we have a better grasp of the information content of neural signals, the applications of neuromodulation in mice must go beyond the simply sensing bio-potentials. Many researchers are interested in studying a particular indication for which there are established readouts such as inflammatory markers or other biomolecules. Even these approaches are limited in their application because of the low volume of blood in mice, sometimes allowing only a single assessment, at the terminal end of an experiment. This points to the need to be able to sense biomolecules in real-time, to better understand the effects of electrical stimulation (or another intervention) on a particular target of interest.

A group of sensing approaches that are well suited to meet this need are electroanalytical techniques that use a potentiostat. These approaches include amperometry, cyclic voltammetry, and impedance spectroscopy. In most cases a potentiostat and its associated electronic circuits can be implemented with only minor design changes to the electrical stimulation and recording electronics already found on many neuromodulation devices. Potentiostat-based electroanalytical techniques sense changes in current between electrodes due to changes in the composition of the solution and how it interacts with the electrodes. Sensor specificity can be achieved using intrinsic characteristics of the electrochemical reaction occurring at the electrodes, and example of this is fast scan cyclic voltammetry (FSCV) used to detect targets based on specific oxidation and reduction kinetics. Another approach to achieve specificity is from the engineered functionalization of the electrode surface, common approaches include the use of target specific enzymes, antibodies, or aptamers (McConnell et al., 2020). FSCV has been used to detect biomolecules in vivo (Wightman, 2006) including catecholamines in whole blood (Nicolai et al., 2017) and in the heart (Chan et al., 2020). Amperometry and impedance spectroscopy using functionalization can be used to sense other biomolecules such as glucose (Kvist et al., 2006) and other metabolic and inflammatory markers (Sánchez-Tirado et al., 2020; Gray et al., 2019; Filik & Avan, 2020; Li et al., 2017). Electroanalytical approaches require few components apart from the electronics and electrodes making them an appealing approach for miniaturization. Wireless FSCV systems have already been demonstrated (Dorta-Quiñones et al., 2016), and future development is likely to bring these capabilities to chronically implantable systems for in-vivo measurement.

## Conclusion

The utility of mouse models in BEM will be greatly improved by the development of wireless closed-loop neuromodulation systems small enough for implantation in mice. In addition to advances in BEM, such devices will enable new basic discoveries for neuroscience and other disciplines. Though there are numerous technical challenges, they appear to be surmountable and recent progress is encouraging, but the motivators must be put into place in the form of research support or dedicated collaborative development programs. The broad skills set required to build these devices requires that a systems-level design approach be utilized, and devices must undergo extensive long-term testing and validation so that they can be adopted by interested researchers without engineering expertise. Much of the technological advancement in this area has come from academic labs which have focused on pushing the envelope in particular areas. Greater participation from industry may be needed to reach widescale adoption since the university research model is not ideal for product development and mass production.

Beyond the technical engineering challenges, little is known about the biological impact of long-term implants in mice. Recent studies indicate that the presence of a chronically implanted vagus nerve electrode does not have significant detrimental effects on physiological or immune markers (Mughrabi et al., 2021), but the presence of the device itself has potentially unknown effects on the health and behavior of the animal. Some key metrics to consider are the impact of the implant on inflammatory markers, organ health, and behavior. It will be important to understand these aspects as research moves forward because the implant itself should not impose a bias on the outcomes of experiments. These remain important open questions.

## Data Availability

Not applicable.

## Abbreviations

BEM: Bioelectronic Medicine
CIC: Charge injection capacity
CNS: Central nervous system
ECG: Electrocardiogram
EMG: Electromyogram
FBR: Foreign body response
FDA: Food and Drug Administration
FSCV: Fast scan cyclic voltammetry
IACUC: Institutional Animal Care and Use Committee
LCP: Liquid crystal polymer
PEDOT: Poly (3,4-ethylenedioxythiophene)
PNS: Peripheral nervous system
SAR: Specific absorption rate
VNS: Vagus nerve stimulation
WVTR: Water vapor transmission rate
